# Infectious disease physician characteristics and prescription of meropenem in the hospital

**DOI:** 10.1017/ash.2023.193

**Published:** 2023-07-17

**Authors:** Douglas Challener, John O’Horo, Aaron J. Tande

**Affiliations:** 1 Division of Public Health, Infectious Diseases and Occupational Medicine, Mayo Clinic, Rochester, MN, USA; 2 Division of Pulmonary and Critical Care Medicine, Mayo Clinic, Rochester, MN, USA

## Abstract

**Objective:**

Physician characteristics may be correlated with medical treatment decisions and patient outcomes. This study examined the correlations between characteristics of infectious disease (ID) physicians and the use of the restricted antimicrobial meropenem.

**Design:**

This was a retrospective cohort study following 27 attending ID physicians for 5 years at a large academic medical center.

**Methods:**

All inpatient ID clinical encounters between 2013 and 2018 were assessed for physician and patient characteristics, including patient Charlson Comorbidity Index, patient sex, ID service seeing the patient, physician career stage, physician training location, and physician sex. Adjusted and unadjusted odds ratios were calculated for the receipt of meropenem on the same day as an ID clinical note.

**Results:**

Between 2013 and 2018, meropenem was administered on the same day as 9046 (11.1%) of 81,787 inpatient ID encounters. After adjustment for patient and practice-specific factors, physician career stage was associated with administration of meropenem. Patients seen by mid-career and late-career ID physicians were more likely to receive meropenem than those seen by early-career physicians (aOR 1.22 95% confidence interval [CI 1.13–1.31 and aOR 1.17 95% CI 1.10–1.25, respectively).

**Conclusions:**

ID provider characteristics may help target future antimicrobial stewardship program interventions.

## Introduction

Physician characteristics such as sex and age have been shown to be correlated with clinical treatment decisions and patient outcomes. Studies in both medical and surgical specialties have revealed significant differences in practice patterns between newly trained physicians and late-career physicians.^1^ Several studies suggest experienced physicians may provide medical care that is lower quality than their younger peers, sometimes resulting in worse outcomes.^2–4^ In contrast, surgical outcomes may improve with increasing clinical experience.^5^ Other studies have failed to show that changes in physician age are associated with meaningful differences in clinical outcomes.^6^ In the field of antimicrobial stewardship, there may be an association with physician career stage and duration of antibiotic prescription, but associations of physician-specific characteristics on antimicrobial prescription have not yet been investigated among infectious disease (ID) specialists.^7,8^ These factors for practice variation may be rich targets for stewardship interventions, as they are not driven by patient or clinical variables.

Hospital antimicrobial stewardship programs (ASPs) often implement antibiotic restriction policies in an effort to preserve efficacy of important and often last-line antibiotics.^9–12^ Additionally, there has been recent interest in the use of peer comparison as a tool for modification of individual prescription practices.^13^ Mayo Clinic in Rochester, Minnesota, has had restriction policies in place to limit the use of the carbapenem class of antibiotics. Primary treatment teams at Mayo Clinic can use these restricted antibiotics with approval from the ASP if they meet predetermined criteria, or if approved by an ID faculty physician, who rotate on the ASP service on a weekly basis. If an ID service is actively following a patient, the ASP will defer treatment decisions to the consulting service. This study aims to explore physician-specific characteristics and their potential impact on the receipt of a restricted antimicrobial in the hospital.

## Methods

We systematically identified inpatient ID clinical notes (initial consultations, follow-up progress notes, or progress sign-off notes) within the electronic health record (EHR) authored between January 1, 2013 and January 1, 2018. Next, we queried the medication administration record (MAR) for administration of meropenem (our institution’s inpatient carbapenem of choice at the time of this study) on the same day as an ID clinical note. A similar query for administration of piperacillin-tazobactam and cefepime was performed to provide interpretive context for the use of nonrestricted broad-spectrum antimicrobial agents. Patient data including age, sex, and comorbid conditions at the time of the encounter were obtained from the EMR. The Charlson Comorbidity Index (CCI) for each patient was calculated using international classification of diseases (ICD) codes. Formulary restriction status of meropenem at the time of each note was also collected. Patients were managed on the following ID subspecialty services: general, solid organ transplant, hematology/oncology/bone marrow transplant, orthopedics, and intensive care unit. Encounters were processed to remove notes from fellows who became faculty (while keeping their notes after faculty appointment). Notes with incorrect subspecialty attributions were also removed (ie, labeled as being general ID from a provider who never practiced on the general service). Notes authored by two physicians with limited, low-volume practice were removed to improve the power of the study (Supplemental Figure 1). One physician in our group practice opted out of the study, and their data were removed.

We retrieved physician date of graduation from medical school from their publicly available institutional profile website. The experience level of the consultant at the time of each encounter was calculated as the time since graduation from fellowship. We placed physician experience into three categories representing early career (less than 10 years since fellowship graduation), mid-career (between 10 and 20 years since fellowship graduation), and late career (20 or more years since fellowship graduation). These definitions are similar to those published by the United States Office of Personnel Management.^14^ Location of fellowship training was classified as Internal if it was performed at our institution or External if it was performed at a different institution. Odds ratios predicting that a patient would receive meropenem on the encounter date using both patient and physician characteristics as predictors were calculated with adjustment for all covariables. All analysis was conducted using R version 4.1.2.^15^ The Comorbidity and Tidyverse R packages were used.^16,17^ The Mayo Clinic Institutional Review Board (IRB) deemed this study exempt from the need for IRB approval and waived the need for patient informed consent (ID 22-004850).

## Results

Between January 1, 2013 and January 1, 2018, there were 81,787 documented inpatient encounters in our ID division from 27 different attending physicians across 5 subspecialty ID consult services. Twenty of the 27 physicians split their time across multiple services. There were 17,418 encounters of the early-career physicians (9 physicians), 29,280 of the mid-career physicians (12 physicians), and 35,089 encounters for the late-career physicians (12 physicians). Meropenem was administered on the same day as 9046 ID encounters (11.1%). Patients who received meropenem on the same day of an ID encounter differed from those who did not in CCI (CCI of 5.9 vs 6.3 in those receiving meropenem, *P* < .01). There were also provider-level differences in experience, training location, and ID subspecialty service (Table 1).

**Table 1.** Odds of meropenem administration by patient and physician characteristics

Characteristic	OR (95%)	*P*	OR (95%) Adjusted	*P*
**Charlson Comorbidity Index (age-adjusted)**	1.03 (1.02, 1.03)	<.01	1.01 (1.00, 1.02)	<.01
**Patient sex**				
Female	Reference		Reference	
Male	0.98 (0.93, 1.02)	.27	0.98 (0.94, 1.03)	.45
**Meropenem restriction status at the time of encounter**				
Not restricted	Reference		Reference	
Restricted	0.87 (0.83, 0.91)	<.01	0.90 (0.86, 0.95)	<.01
**Infectious disease service**				
General	Reference		Reference	
Intensive care unit	2.26 (2.08, 2.46)	<.01	2.23 (2.05, 2.43)	<.01
Orthopedics	1.02 (0.93, 1.11)	.72	1.02 (0.93, 1.12)	.70
Hematology-oncology and bone marrow transplantation	4.76 (4.41, 5.15)	<.01	4.73 (4.37, 5.13)	<.01
Solid organ transplantation	3.44 (3.16, 3.74)	<.01	3.25 (2.98, 3.55)	<.01
**Physician career stage** (years since fellowship completion)				
Early (<10 years)	Reference		Reference	
Middle (10–20 years)	0.92 (0.87, 0.97)	<.01	1.22 (1.13, 1.31)	<.01
Late (>20 years)	0.87 (0.82, 0.97)	<.01	1.17 (1.10, 1.25)	<.01
**Physician fellowship location**				
External fellowship	Reference		Reference	
Internal fellowship	1.39 (1.25, 1.54)	<.01	1.20 (1.08, 1.34)	<.01
**Physician medical school location**				
US Graduate	Reference		Reference	
International medical graduate	0.70 (0.67, 0.74)	<.01	0.83 (0.78, 0.89)	<.01
**Physician sex**				
Female	Reference		Reference	
Male	1.18 (1.11, 1.24)	<.01	1.06 (0.99, 1.13)	.11

The odds of receipt of meropenem on a given day (Table 1) showed highest increase in those being seen on the HO-BMT service (aOR 4.73; 95% CI 4.37–5.13). This population was also more likely to receive a nonrestricted broad-spectrum antibiotic (ie, piperacillin-tazobactam or cefepime; Supplemental Table 1), albeit less so (aOR 2.00; 95% CI 1.91–2.09). Patients receiving meropenem were also more likely to have been seen by physicians who trained in internal fellowships (aOR 1.20; 95% CI 1.08–1.34).

Career stage of the ID specialist was significantly associated with the odds of a patient to receive meropenem. Patients seen by mid-career ID physicians were more likely to receive meropenem than those seen by early-career physicians (aOR 1.22; 95% CI 1.13–1.31). Likewise, patients seen by late-career physicians were more likely to receive meropenem on the same day compared to those seen by early-career physicians (aOR 1.17; 95% CI 1.10–1.25). This effect was not seen with other broad-spectrum antibiotics (piperacillin-tazobactam or cefepime) where the odds of a patient receiving the drug decreased with physician career stage (aOR 0.99 for mid-career physicians, and 0.91 for late-career physicians, compared to early-career physicians).

Antimicrobial restriction was inversely associated with antibiotic receipt. Meropenem became a restricted antimicrobial agent on June 1, 2014. Prior to this restriction, 12.0% of patients received meropenem on the same day as an ID consultation, following the restriction, this dropped to 10.6% (*P* < .01). This effect was driven by the hematology/oncology service where the prescription rates decreased from 24.0% to 19.3% (*P* < .01). Most other services did not see statistically different changes in prescription practice (Supplemental Table 2). Supplemental Figure 2 also illustrates a crossing-over of utilization of piperacillin-tazobactam and cefepime in early 2017 likely reflecting concerns over the potential nephrotoxicity of vancomycin and piperacillin-tazobactam and the transitions away from this empiric regimen.

## Discussion

ID physician career stage is associated with use of meropenem on the same day as consultation. After adjusting for patient factors including CCI as well as physician factors including subspeciality consulting service, and restriction status of the drug, career stage was associated with differential prescribing of meropenem. This effect was most pronounced in the mid-career physicians though late-career physicians also utilized more than their early-career peers. In contrast, the adjusted utilization of piperacillin-tazobactam or cefepime was lower for both mid- and late-career physicians compared to early-career physicians. This suggests a unique effect of meropenem not seen in similarly broad-spectrum antibiotics of other classes.

As in other fields, practice patterns in IDs appear to change over the course of one’s career. This may be a result of many factors, including experience, comfort with uncertainty, and concern with adherence to ASP restrictions. The size of the increased use of meropenem in more experienced physicians was much lower than the magnitude of the effect of the subspecialty consulting service on which the physician was rotating. This suggests that in addition to physician characteristics, patient factors are strong driving forces for antimicrobial selection, and these patient populations provide opportunities for ASP interventions.

Training location was also associated with utilization of meropenem with physicians who received undergraduate medical training within the United States and those who received fellowship training within our program seeing more patients who received meropenem when adjusting for other factors. Previous studies have also noticed patterns between training location and antimicrobial prescription practices.^18^ Practice patterns observed during medical training may be assimilated by trainees. This illustrates the importance of attending physicians modeling antimicrobial stewardship principles for learners.

This study benefits from a large data set from a large academic ID division with wide variety of patient and physician characteristics. The availability of and adjustment for physician and patient characteristics is another strength of this study. Members of the division were spread across different focus groups, location of training, and career experiences allowing for comparison. The study is limited by its retrospective nature and data from a single site. There are many host and pathogen factors that may influence the physician to recommend meropenem but may not be accounted for in the current approach. Additionally, there are practical variables such as number of daily consultations and number of fellows on service that were not studied. Adjustment for CCI was based on ICD codes which are known to only be a crude indicator of medical conditions. Limitation is that utilization of various antimicrobials on the same day as a consultation note may not reflect the recommendations of the consulting physician. Lastly, by excluding a division member who opted out, the results may be skewed.

## Conclusions

In this exploratory analysis of a large academic ID division, physician-specific factors of the consulting specialist were found to be associated with utilization of meropenem. These factors could be considered when designing ASP interventions. A future study could examine providers practice patterns longitudinally over many years to further evaluate the impact that experience has on an individual level.

## Supplementary material

For supplementary material accompanying this paper visit http://doi.org/10.1017/ash.2023.193. click here to view supplementary material
