# Supplementary material for: Infectious disease physician characteristics and prescription of meropenem in the hospital
Source: Antimicrob Steward Healthc Epidemiol. 2023 Jul 17;3(1):e126. doi: 10.1017/ash.2023.193 (PMC10390666; doi:10.1017/ash.2023.193)
Supplement: Supplementary file 1 [file ashsup.zip › S2732494X23001936sup001.docx]

**Supplemental Table 2** – Number of Encounters on the Same Day as Meropenem Administration Before and After Restriction

|  | Meropenem not restricted | Meropenem Restricted |
| --- | --- | --- |
| n | 23738 | 58049 |
|  |  |  |
|  |  |  |
| **Received Meropenem** | 2862 (12.0%) | 6186 (10.6%) |
| Intensive Care Unit | 512 (10.6%) | 1375 (11.1%) |
| Orthopedics | 330 (5.7%) | 708 (5.1%) |
| Hematology-Oncology and Bone Marrow Transplantation | 1161 (24.0%) | 2258 (19.3%) |
| General | 258 (5.8%) | 607 (5.0%) |
| Solid Organ Transplantation | 601 (15.8%) | 1236 (15.8%) |
|  |  |  |
