# Supplementary material for: Infectious disease physician characteristics and prescription of meropenem in the hospital
Source: Antimicrob Steward Healthc Epidemiol. 2023 Jul 17;3(1):e126. doi: 10.1017/ash.2023.193 (PMC10390666; doi:10.1017/ash.2023.193)
Supplement: Supplementary file 1 [file ashsup.zip › S2732494X23001936sup002.docx]

| **Characteristic** | **OR (95%)** | **p** | **OR (95%) Adjusted*** | **p** |
| --- | --- | --- | --- | --- |
| **Charlson Comorbidity Index (Age Adjusted)** | 1.02 (1.01, 1.02) | <0.01 | 1.02 (1.01, 1.02) | <0.01 |
| **Patient Sex** |  |  |  |  |
| Female | reference |  | reference |  |
| Male | 1.08 (1.05, 1.11) | <0.01 | 1.08 (1.05, 1.11) | <0.01 |
|  |  |  |  |  |
| **Meropenem restriction status at time of encounter** |  |  |  |  |
| Not restricted | reference |  | reference |  |
| Restricted | 1.19 (1.16, 1.23) | <0.01 | 1.17 (1.14, 1.21) | <0.01 |
|  |  |  |  |  |
| **Focus Group** |  |  |  |  |
| General | reference |  | reference |  |
| Intensive Care Unit | 1.70 (1.63, 1.77) | <0.01 | 1.70 (1.63, 1.78) | <0.01 |
| Orthopedics | 1.19 (1.14, 1.24) | <0.01 | 1.23 (1.18, 1.28) | <0.01 |
| Hematology-Oncology and Bone Marrow Transplantation | 2.01 (1.92, 2.10) | <0.01 | 2.00 (1.91, 2.09) | <0.01 |
| Solid Organ Transplantation | 0.99 (0.94, 1.03) | 0.61 | 0.98 (0.93, 1.03) | 0.4 |
|  |  |  |  |  |
| **Physician Career Stage** (years since fellowship completion) |  |  |  |  |
| Early (<10 y) | reference |  | reference |  |
| Middle (10-20 y) | 0.80 (0.77, 0.83) | <0.01 | 0.99 (0.94, 1.03) | 0.5 |
| Late (>20 y) | 0.81 (0.78, 0.84) | <0.01 | 0.94 (0.90, 0.97) | <0.01 |
|  |  |  |  |  |
| **Physician Fellowship Location** |  |  |  |  |
| External Fellowship | reference |  | reference |  |
| Internal Fellowship | 0.89 (0.84, 0.94) | <0.01 | 0.91 (0.85, 0.97) | <0.01 |
|  |  |  |  |  |
| **Physician Medical School Location** |  |  |  |  |
| United States Graduate | reference |  | reference |  |
| International Medical Graduate | 0.92 (0.89, 0.95) | <0.01 | 0.96 (0.92, 1.00) | 0.07 |
|  |  |  |  |  |
| **Physician Sex** |  |  |  |  |
| Female | reference |  | reference |  |
| Male | 0.98 (0.95, 1.01) | 0.23 | 1.02 (0.98, 1.06) | 0.4 |

Supplemental Table 1 – Odds of Cefepime or Piperacillin-Tazobactam Administration by Patient and Physician Characteristics
